# Analytical and clinical validity of wearable, multi-sensor technology for assessment of motor function in patients with Parkinson’s disease in Japan

**DOI:** 10.1038/s41598-023-29382-6

**Published:** 2023-03-14

**Authors:** Genko Oyama, Maximilien Burq, Taku Hatano, William J. Marks, Ritu Kapur, Jovelle Fernandez, Keita Fujikawa, Yoshihiko Furusawa, Keisuke Nakatome, Erin Rainaldi, Chen Chen, King Chung Ho, Takashi Ogawa, Hikaru Kamo, Yutaka Oji, Haruka Takeshige-Amano, Daisuke Taniguchi, Ryota Nakamura, Fuyuko Sasaki, Shinichi Ueno, Kenta Shiina, Anri Hattori, Noriko Nishikawa, Mayu Ishiguro, Shinji Saiki, Ayako Hayashi, Masatoshi Motohashi, Nobutaka Hattori

**Affiliations:** 1 Department of Neurology, Juntendo University Faculty of Medicine, 2-1-1, Hongo, Bunkyo-Ku, Tokyo, 113-8421 Japan; 2 Verily Life Sciences, 269 East Grand Avenue, South San Francisco, CA USA; 3 Takeda Pharmaceutical Company Limited, 2 Chome-1-1 Nihonbashihoncho, Chuo-Ku, Tokyo, 103-0023 Japan

**Keywords:** Parkinson's disease, Biomarkers

## Abstract

Continuous, objective monitoring of motor signs and symptoms may help improve tracking of disease progression and treatment response in Parkinson’s disease (PD). This study assessed the analytical and clinical validity of multi-sensor smartwatch measurements in hospitalized and home-based settings (96 patients with PD; mean wear time 19 h/day) using a twice-daily virtual motor examination (VME) at times representing medication OFF/ON states. Digital measurement performance was better during inpatient clinical assessments for composite V-scores than single-sensor–derived features for bradykinesia (Spearman |r|= 0.63, reliability = 0.72), tremor (|r|= 0.41, reliability = 0.65), and overall motor features (|r|= 0.70, reliability = 0.67). Composite levodopa effect sizes during hospitalization were 0.51–1.44 for clinical assessments and 0.56–1.37 for VMEs. Reliability of digital measurements during home-based VMEs was 0.62–0.80 for scores derived from weekly averages and 0.24–0.66 for daily measurements. These results show that unsupervised digital measurements of motor features with wrist-worn sensors are sensitive to medication state and are reliable in naturalistic settings.

**Trial Registration:** Japan Pharmaceutical Information Center Clinical Trials Information (JAPIC-CTI): JapicCTI-194825; Registered June 25, 2019.

## Introduction

Parkinson’s disease (PD) is a neurodegenerative movement disorder associated with diverse motor and non-motor signs and symptoms that change frequently and vary widely between patients^1,2^. The core motor symptoms associated with PD are bradykinesia, tremor, rigidity, and postural instability, which in part contribute to gait disturbance, one of the early hallmark features of PD^1,3^. Levodopa (combined with an aromatic L-amino acid [DOPA] decarboxylase inhibitor) is the most common treatment for PD motor symptoms^4^, but treatment optimization is challenging. After several years of treatment, many patients experience “wearing off”—where symptoms re-emerge or worsen before a dose is due—and may develop dyskinesias (involuntary or erratic movements) in response to excess, or fluctuations in, levodopa concentration^1,5^.

Management of PD symptoms and identification of disease-modifying compounds require sensitive and reliable measurements of disease status. Treatment customization and tracking of disease progression are typically based on clinical assessments such as the Movement Disorder Society-sponsored revision of the Unified Parkinson’s Disease Rating Scale (MDS-UPDRS)^6^, which incorporates patient-reported outcomes regarding non-motor symptoms, activities of daily living, and motor complications such as wearing off and dyskinesia. However, assessments of motor symptoms are based on subjective clinician ratings and, because they are performed in a clinic, can only capture a “snapshot” of the motor function that patients experience in their daily lives. Patients can also be asked to complete a medication or symptom diary, but by nature this information can be incomplete and/or inaccurate^7^. Because current assessments are not designed for continuous monitoring of signs and symptoms, treatment customization usually requires repeated in-clinic visits over several months to titrate drug levels. These visits impose an additional burden on patients and caregivers and are limited to those patients who are able to visit major medical centers. In addition, subjective assessments have an inherent variance that makes it difficult to detect subtle changes in disease progression, particularly over the 1–2-year time frame of most clinical studies in PD, and do not give an accurate picture of disease trajectory over many years^8^. Therefore, there is a need for quantitative, objective measures that can be acquired more frequently and outside of clinical settings.

New technologies that enable continuous and objective measurement of motor signs and symptoms are currently being evaluated for use in patients with PD and are expected to overcome the challenges associated with in-clinic symptom monitoring and treatment optimization^9–11^. Preliminary findings suggest that wearable devices as well as smartphone applications can be adopted in remote and home-based settings to assist with identification of motor fluctuations, PD symptom onset and monitoring, and drug titration^12–17^. However, these studies have largely focused on comparing in-clinic measurements with clinical ratings^18^, and most home-based studies have been conducted with small numbers of patients^12–14^ or included a limited number of motor signs and symptoms^13,15,16^.

**Figure 1:**
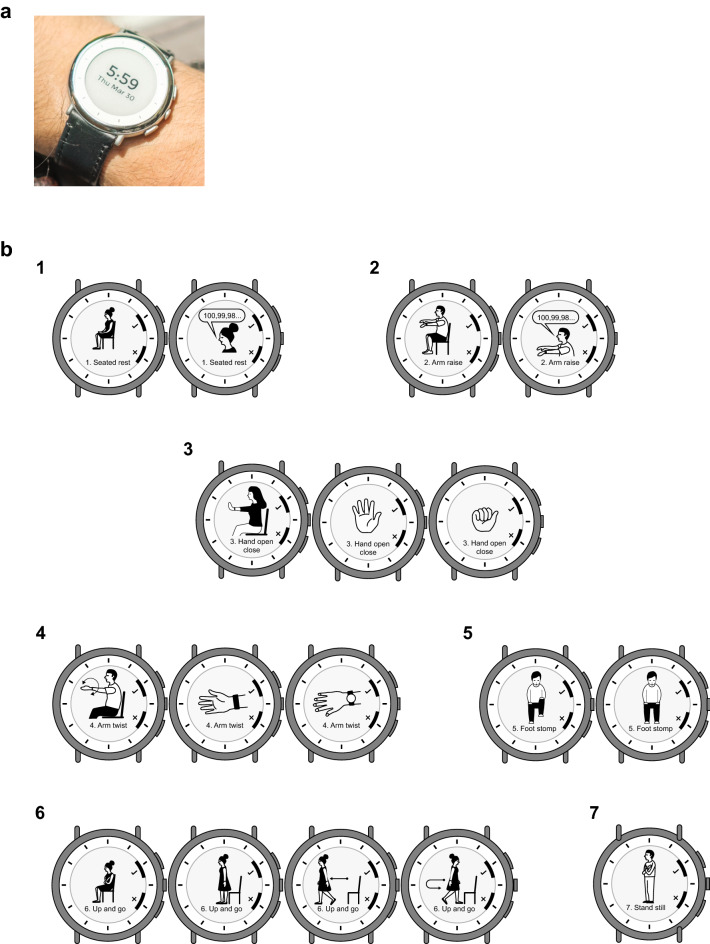
Study Watch and watch face showing the virtual motor examination. (**a**) Study Watch worn on the wrist and (**b**) Study Watch face showing the virtual motor examination. The virtual motor examination comprised seven structured motor tasks, which were programmed into the smartwatch and completed by patients in order from task 1 through task 7. Tasks 1 (seated rest/rest tremor), 2 (arm raise/postural tremor), 3 (hand opening/closing), 4 (arm twist/pronation/supination), and 5 (foot stomping) were completed seated. Tasks 6 (up and go) and 7 (stand still/postural stability) required the patient to stand.

The Verily Study Watch (Verily Life Sciences LLC, South San Francisco, CA, USA, Fig. 1a) is a multi-sensor wrist-worn smartwatch device that enables passive data capture throughout the course of daily life and incorporates a task-based virtual motor examination (VME, Fig. 1b) for contextualized data collection in remote settings. The VME comprises seven tasks that were designed to assess various domains of motor symptoms in PD: rest and postural tremor (tasks 1, 2), upper extremity bradykinesia through repeated hand opening and closing and pronation supination (tasks 3, 4), lower extremity bradykinesia through foot stomping (task 5), gait (task 6), and postural sway (task 7). The smartwatch has been assessed in Dutch patients with early-stage PD using a weekly-based VME over 1 year (Personalized Parkinson Project [PPP])^19^. The current study, which was conducted in Japan, aims to reveal the usefulness of the smartwatch for precise monitoring of PD motor function and was designed to obtain insight into the clinical utility of the device for broader use in inpatient and home-based settings in patients with PD from a different population and healthcare system, and using a high-frequency VME. This report provides insights into patient behavior and acceptance of wearing the smartwatch for up to 23 h/day while undertaking a twice-daily VME during hospitalization and during a 1-month home-based assessment. In addition, information on the analytical and clinical validity of the smartwatch when used in a well-controlled inpatient setting and in naturalistic, home-based settings is described.

## Results

### Demographic and baseline clinical characteristics

**Figure 2:**
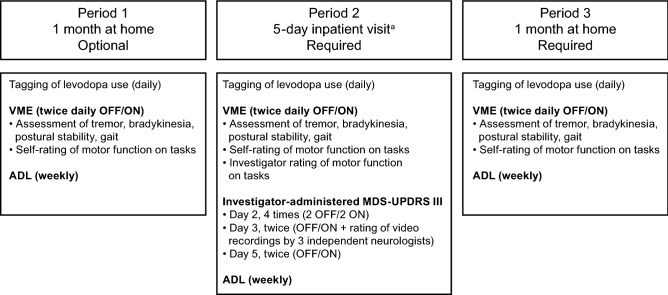
Study design. Period 1 was optional to accommodate the most flexible schedule for patients. Patients could enroll in the study before Period 1 in an outpatient clinic or immediately before or during Period 2. At enrollment, patients were screened for eligibility, provided written informed consent, received the smartwatch, and underwent baseline study assessments. During Period 2, patients were washed out of all concomitant Parkinsonian medications. At the final outpatient clinic visit, patients underwent final assessments and returned the smartwatch. The duration of each period may have varied from the specified durations due to the convenience of having the device dispensed and returned at the study site, the timing of the patient’s scheduled visit, and the length of the hospital stay depending on their individual treatment needs; all available data were included in the analysis regardless of the duration of each period. The VME, comprising seven structured motor tasks, was conducted at scheduled times representing relatively poor symptom control (when medication was wearing off [OFF]) and good symptom control (when medication was working well [ON]). ^a^During the 5-day inpatient period, patients could undergo an optional 2 days of assessments, which were not included in the study. *ADL* activities of daily living, *MDS-UPDRS* Movement Disorder Society-sponsored revision of the Unified Parkinson’s Disease Rating Scale, *VME* virtual motor examination.

**Figure 3:**
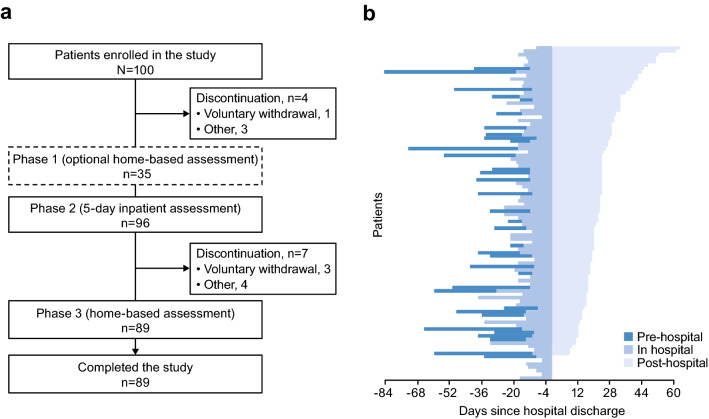
Patient flow. (**a**) Study flow diagram and (**b**) individual patient participation. Patients wore the smartwatch during the 1-month pre- or post-hospitalization periods and during the 5-day inpatient assessment.

Of the 100 patients enrolled, 96 received the smartwatch, had valid VMEs, and were included in the full analysis set (Figs. 2 and 3). Of these, 35 participated in an optional 1-month home-based pre-hospitalization assessment (Period 1). All 96 patients participated in the 5-day inpatient assessment (Period 2), and 84 patients participated in the 1-month home-based post-hospitalization assessment (Period 3) (Supplementary Table 1).

**Table 1:** Patient demographics and characteristics.

Variable	Patients (N = 96)
Age, years	62.3 (8.5)
Age categories, n (%)	
< 40 years	2 (2.1)
40 to < 65 years	49 (51.0)
65 to < 75 years	41 (42.7)
≥ 75 years	4 (4.2)
Sex, n (%)	
Male	44 (45.8)
Female	52 (54.2)
Time from first onset of Parkinson’s disease symptoms, years	12.5 (5.8)
Caregiver availability, n (%)	63 (65.6)
History of deep brain stimulation	8 (8.3)
Hoehn and Yahr score^a^	2.8 (0.9)
Stage 1, n (%)	0 (0.0)
Stage 2, n (%)	41 (43.6)
Stage 3, n (%)	38 (40.4)
Stage 4, n (%)	5 (5.3)
Stage 5, n (%)	10 (10.6)
MDS-UPDRS Part I total score	12.9 (5.7)
MDS-UPDRS Part II total score	16.9 (6.8)
MDS-UPDRS Part III total score^b^	46.1 (21.4)
Part III bradykinesia^b^	14.0 (6.5)
Part III postural instability^b^	1.4 (0.8)
Part III upper extremity tremor^b^	2.2 (2.6)
MDS-UPDRS Part IV total score	8.1 (3.5)
MDS-UPDRS consensus-UPDRS total score^c^	88.9 (23.2)
MDS-UPDRS mean-UPDRS total score^d^	84.7 (21.7)
PDQ-39 summary index	33.9 (17.3)
Schwab and England ADL scale	73.5 (12.4)

Data are mean (SD) unless reported otherwise.

^a^Assessed in Period 2, Day 3 of inpatient assessment, OFF state.

^b^Assessed in Period 2, Day 2 of inpatient assessment, OFF state.

^c^Consensus scores for the MDS-UPDRS Part III were the average of three neurologist ratings (OFF medication state).

^d^Mean MDS-UPDRS Part III scores were calculated from scores on Days 2, 3, and 5 of the inpatient assessment (ON medication state).

*ADL* Activities of Daily Living, *MDS-UPDRS* Movement Disorder Society-sponsored revision of the Unified Parkinson’s Disease Rating Scale, *PDQ-39* 39-item Parkinson’s Disease Questionnaire.

At enrollment, 54% (52/96) of patients were female, and the mean (standard deviation [SD]) age was 62.3 (8.5) years (Table 1). Patients had experienced PD symptoms for a mean of 12.5 (5.8) years, 65.6% (63/96) had a caregiver, and all patients were taking Parkinsonian medications, including levodopa. On Day 3 of the inpatient assessment, most patients were at Hoehn and Yahr Stage 2 (43.6%, 41/96) or Stage 3 (40.4%, 38/96) during the OFF state.

Among the 727 MDS-UPDRS Part III assessments, 25% (183/727) were conducted when dyskinesia was present. According to investigators, dyskinesia interfered with ratings in 21.3% (39/183) of assessments (i.e., 5.4% [39/727] of all MDS-UPDRS Part III assessments).

### Patient engagement with the device

Most patients (90.6% [87/96]) preferred to wear the smartwatch on their left arm, and 74.2% (69/93) of patients wore the smartwatch on their most affected side. Approximately half (50.5% [47/93]) of patients reported that their left side was most affected, 25.8% (24/93) reported that their right side was most affected, and 23.7% (22/93) reported that both sides were affected equally.

The mean (SD) percentage of VMEs completed (completion of all seven tasks) was 60.9% (26.7%) for the whole study, 66.2% (27.3%) for Period 1, 68.3% (19.2%) for Period 2, and 65.0% (28.9%) for Period 3. The mean (SD) percentage of patients who completed at least two VMEs per day in Period 3 was 56.0% (7.3%). Most VMEs were completed within 10 min, and there was no major difference in the number of VMEs completed between the ON and OFF states (Supplementary Fig. 1). Of the VME tasks that were not completed, “up and go” (task 6) and “postural stability” (task 7) were most frequently skipped, and skipping behavior did not appear to differ between ON and OFF medication states (Supplementary Fig. 1). Patients with Hoehn and Yahr Stage 5 accounted for a large proportion of the skipped tasks 6 and 7. Of the 437 assessments of skipped task 6, 108 (24.7%) were by patients with Hoehn and Yahr Stage 5 and 35 (8.0%) were by patients with Hoehn and Yahr Stage 4. Of the 397 assessments of skipped task 7, 103 (25.9%) were by patients with Hoehn and Yahr Stage 5 and 15 (3.8%) were by patients with Hoehn and Yahr Stage 4.

Overall, 72.8% of respondents (67/92) reported their satisfaction with smartwatch comfort as satisfied (52.2%) or neutral (20.7%), and most patients found the smartwatch easy to use (Supplementary Fig. 2). The main reason for not wearing the watch every day was that patients forgot to put it on (33.3%, 10/30 respondents), and 62.0% (57/62) of respondents indicated that they would not wear the watch for longer than the study period. However, questions on reasons for not wanting to wear the smartwatch were not included in the survey.

### Analytical validity of digital measurements

**Figure 4:**
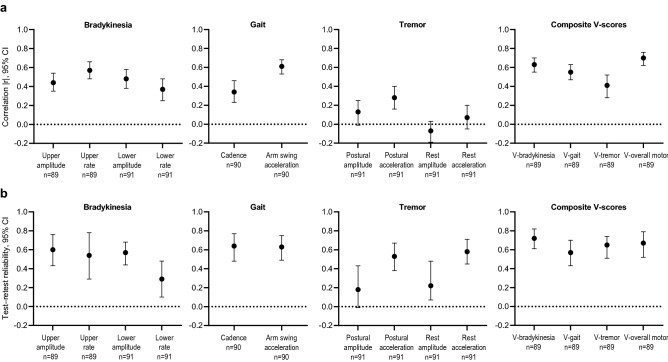
Analytical validity of digital measurements from single-sensor–derived features and composite V-scores. (**a**) Spearman rank correlation between MDS-UPDRS Part III sensor scores and neurologist-rated consensus scores on Day 3 and (**b**) test–retest reliability on Day 2 of the inpatient assessment.^a,b^ Consensus scores for the MDS-UPDRS Part III examination on Day 3 of the inpatient assessment were calculated using an in-person rating from videotaped ratings from three neurologists. The averages of all scores for the OFF and ON states on Day 3 were combined for each measure. ^a^Spearman rank correlation coefficients are plotted as absolute values; original values are plotted for coefficients where the 95% CI crosses the 0 line. Correlation was considered weak for coefficients < 0.3, moderate for coefficients 0.3–0.6, and strong for coefficients > 0.6. ^b^Test–retest reliability was computed from MDS-UPDRS Part III sensor scores on Day 2, in which the MDS-UPDRS examination was administered twice within a short period of time; test–retest reliability was considered poor for ICCs < 0.5, average for ICCs 0.5–0.75, good for ICCs > 0.75–0.9, and excellent for ICCs > 0.9. *CI* confidence interval, *ICC* intraclass correlation coefficient, *MDS-UPDRS* Movement Disorder Society-sponsored revision of the Unified Parkinson’s Disease Rating Scale, *V-score* machine-learned composite sensor scores for each motor feature.

In general, correlation between MDS-UPDRS Part III digital measurements and corresponding neurologist-rated consensus scores, and test–retest reliability of digital measurements, were better for composite “V-scores” for each motor feature compared with single-sensor–derived features and were best for the overall motor V-score (Fig. 4, Supplementary Table 2). For all single-sensor features, correlation coefficients (|r|) were ≤ 0.61 and test–retest reliabilities (intraclass correlation coefficients [ICCs]) were ≤ 0.64. For composite V-scores, correlations and test–retest reliability were better than the single-sensor features for bradykinesia (|r|= 0.63, ICC = 0.72) and tremor (|r|= 0.41, ICC = 0.65), and were not greatly different for gait (|r|= 0.55, ICC = 0.57) (Fig. 4, Supplementary Table 2). For the overall motor V-score, correlation between neurologist-rated consensus scores was 0.70 and test–retest reliability was 0.67.

### Clinical validity of digital measurements: pharmacodynamic biomarker

**Figure 5:**
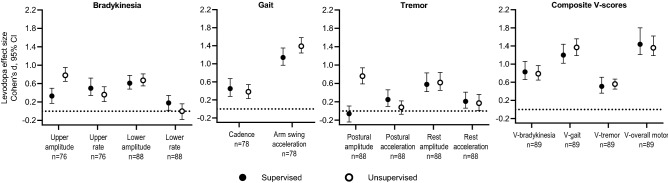
Clinical validity of digital measurements from single-sensor–derived features and composite V-scores as pharmacodynamic biomarkers: levodopa effect sizes calculated using sensor data collected during the supervised MDS-UPDRS Part III and unsupervised VME on Day 3 of the inpatient assessment. Effect sizes were calculated using Cohen’s d. Effect sizes of 0.2 were considered small, 0.5 were considered medium, 0.8 large, and > 1.2 very large. The smartwatch sensor data were collected during the investigator-supervised MDS-UPDRS Part III examination and during the unsupervised VME on Day 3 of the inpatient assessment; the averages of all scores on Day 3 were combined for each measure. *CI* confidence interval, *MDS-UPDRS* Movement Disorder Society-sponsored revision of the Unified Parkinson’s Disease Rating Scale, *V-score* machine-learned composite sensor scores for each motor feature, *VME* virtual motor examination.

The smartwatch digital measurements were sensitive to changes in medication state on Day 3 of the inpatient assessment during the supervised and unsupervised motor examinations. Levodopa effect sizes assessed during the investigator-supervised MDS-UPDRS Part III examination were small to medium for all single-sensor–derived features (irrespective of direction) for bradykinesia (0.18–0.61) and tremor (0.06–0.58), and were medium to very large for gait (0.45–1.14) (Fig. 5, Supplementary Table 3). For the composite V-scores, effect sizes were better for bradykinesia (0.83) and gait (1.2), but not tremor (0.51). There were no major differences in effect sizes for digital measurements collected during the investigator-supervised MDS-UPDRS Part III examination or unsupervised VME (Fig. 5). Levodopa effect sizes for the composite V-scores during the inpatient unsupervised VME were 0.79 for bradykinesia, 1.37 for gait, and 0.56 for tremor.

**Figure 6:**
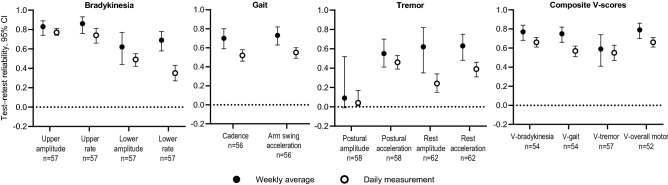
Test–retest reliability of VME single-sensor–derived features and composite V-scores during the post-hospitalization home-based assessment. Test–retest reliabilities are reported for daily measurements and weekly averages of all scores for the OFF and ON states during the post-hospitalization period. Test–retest reliability was considered poor for ICCs < 0.5, average for ICCs 0.5–0.75, good for ICCs > 0.75–0.9, and excellent for ICCs > 0.9. *CI* confidence interval, *ICC* intraclass correlation coefficient, *V-score* machine-learned composite sensor scores for each motor feature, *VME* virtual motor examination.

Test–retest reliabilities during the home-based VME improved when digital measurements were derived from weekly averages rather than daily measurements (Fig. 6, Supplementary Table 4). The week-to-week test–retest reliabilities of all at-home single-sensor–derived features and composite V-scores were average to good. Week-to-week test–retest ICCs for composite V-scores were 0.77 for bradykinesia, 0.75 for gait, and 0.62 for tremor, and were highest (0.80) for the overall motor score.

In general, most digital measurements collected during the home-based VME were weakly correlated with patient self-reported functioning (Parkinson’s Disease Questionnaire-39 [PDQ-39] summary index, Schwab and England Activities of Daily Living [ADL] total score, and MDS-UPDRS Part II ADL scores; Supplementary Table 5). Neurologist-rated MDS-UPDRS Part III consensus scores were weakly or moderately correlated with digital measurements collected during the VME on Day 3 of the inpatient assessment or during the post-hospitalization home-based period (Supplementary Table 6).

## Discussion

This is the first longitudinal study to report increased agreement with clinical ratings, reliability, and ability to discriminate medication status when aggregating digital measurements across motor domains into an overall composite V-score and across time. These digital measurements were obtained using a smartwatch during an unsupervised active test (the VME) in a large number of patients with PD in both inpatient and home-based settings. Consistent with the PPP cohort^19^, in which a lower-frequency VME was assessed in home-based settings for 1 year, these findings showed that smartwatch composite V-scores have good analytical validity and potential for use as pharmacodynamic biomarkers in patients with PD. Although use of wearable devices for the detection of slow involuntary movements or movements of low amplitude that are indicative of the OFF state is challenging, this study suggested that detection of the OFF state may be feasible by combining a VME with passive movement monitoring in an inpatient setting. However, further research is required to confirm the use of digital measurements in home-based settings and as monitoring biomarkers.

Consistent with previous studies^16,19^, digital measurements collected with the smartwatch were able to capture clinically relevant features of a range of PD motor signs and symptoms. Inpatient composite V-scores correlated well with neurologist-rated MDS-UPDRS Part III consensus scores and were more accurate and reliable estimates of bradykinesia, tremor, and overall motor features than single-sensor–derived features. Because of the subjectivity of clinical ratings, which requires clinicians to integrate multiple motor features into a single score, it is expected that aggregation of multiple individual sensor features into a composite V-score would improve correlation with neurologist ratings. In contrast to bradykinesia and tremor, there was little difference in correlation coefficients and test–retest reliabilities between single-sensor–derived features for gait and the composite gait V-score. This finding is consistent with those of the PPP cohort, which showed lower correlation between smartwatch digital measurements and clinically rated gait scores compared with other motor features, even with monthly aggregation of scores^19^. Part of the reason for these observations is that digital measurement of gait with a wrist-worn device predominantly measures upper extremity signs during ambulation, whereas clinical assessments, such as the MDS-UPDRS Part III, include additional factors such as “height of foot lift” and “heel strike while walking.”

In order to assess clinical validity, the principles outlined in the BEST evidentiary framework^20^ were applied to determine whether the sensor-based measurements could capture clinically meaningful changes in signs and symptoms in response to treatment. Findings from the inpatient assessment demonstrated that the smartwatch sensor data were sufficiently sensitive to discriminate between levodopa medication states, when data were collected during the neurologist-supervised MDS-UPDRS Part III examination and when patients conducted the VME without neurologist supervision. At this inpatient assessment, patients underwent a levodopa challenge, where levodopa was administered under controlled clinical conditions during patients’ worst OFF state (12 h from the last levodopa dose) and best ON state (1 h after an intravenous levodopa dose). Thus, this assessment has provided insight into digital measurement of a pharmacodynamic response for the maximum effect of levodopa administration on motor features that could be measured with the current set of digital features from the smartwatch. Measurement of treatment response in home-based settings is expected to be complicated by multiple factors, including reduced time in the OFF state (when medication titration is working well), patient adherence with and accuracy of tagging medication ON and OFF states, and the use of concomitant medications for management of PD motor symptoms. Therefore, the findings from the inpatient assessment will be used to design future studies to assess levodopa effect sizes and treatment response in home-based, naturalistic settings. One of the limitations of this analysis is that the potential confounding effects of dyskinesia on digital measurements of motor features were not assessed. However, findings from a sensitivity analysis (data not shown) suggested that although dyskinesia may have contributed to lower levodopa effect sizes for individual sensor features, there were no major differences in composite V-scores in the overall cohort compared with those without dyskinesia in the inpatient assessment.

Although this study was not designed to validate the use of digital measurements as biomarkers of disease progression or therapeutic intervention in the home-based setting, the results were promising. A key finding from the home-based assessments was that digital measurements from single-sensor–derived features with the twice-daily unsupervised VME were affected by the “noise” that resulted from daily fluctuations in signs and symptoms in a naturalistic setting, but temporal aggregation helped reduce this noise and provide increased reliability over daily measurements. This finding is similar to that of the smartwatch in the PPP cohort, where increased reliability was obtained for monthly or bimonthly aggregation of scores compared with weekly aggregation with a once-weekly VME in the home-based setting^19^.

The patients in this study had a broad range of motor symptoms and experienced at least one troubling motor “OFF” period each day. On average, patients were slightly younger than is typically reported for patients with PD in Japan^2^, which suggests the findings could be representative of a population that is likely to be more familiar with smartwatches. Patients found the smartwatch comfortable and easy to use and were able to wear the smartwatch for most of the day, every day. Although patients were asked to undertake a high-frequency examination program, engagement with the VME was encouraging. Most examinations were completed within 10 min, task duration or task skipped did not appear to be affected by medication status, and 60.9% of the examination tasks were completed during the study. It was not surprising that the most frequently skipped tasks were “up and go” and “postural stability” given that these were the last two tasks in the examination and were the most difficult, especially for patients with balance disturbance during the OFF state. It is impossible to obtain measurements of gait and balance tasks from people with Hoehn and Yahr Stage 4 or 5; however, measurements of upper limb bradykinesia from VME tasks can still be useful in tracking progression of symptoms and intervention-related changes. Additionally, sensor data continuously recorded from free-living states may give insights into sleep behavior, degree of mobility, and cardiovascular-related physiological changes (e.g., heart rate, heart rate variability) that are known to be affected by disease progression. Although patient engagement with the smartwatch was encouraging, many respondents indicated that they preferred not to continue using the smartwatch beyond the study period. However, the question posed in the survey did not explore the reasons for not continuing to wear the smartwatch and in particular did not distinguish between the duration of wear and the high-frequency VME. It is likely that the burden of engaging in an active examination twice daily contributed to patients preferring not to continue wearing the device. Findings from the PPP cohort in the Netherlands^21^ showed that patients with PD will wear the smartwatch for up to 2 years. However, in the PPP cohort, the VME was conducted weekly rather than daily. Together, findings from both studies suggest that high-frequency (e.g., daily) examinations should not extend beyond several months, whereas less frequent examinations (e.g., weekly or monthly) are more suitable for long-term monitoring of disease status and progression in PD. This is especially so given that data from the VME are complemented by continuous data streams passively collected as patients go about their daily lives.

This study is one of the first observational studies to provide real-world information on the use of a smartwatch that enables both passive and prompted task-based data collection in a naturalistic setting. Although no single device can measure all aspects of PD, body-worn sensors that enable simultaneous and passive collection of data are likely to result in higher patient engagement and acceptance than can be achieved with other more interactive sensor modalities^22,23^. Compared with previous studies^14,24–27^, a large number of patients were enrolled and completed the study, and multiple motor features were assessed. Importantly, both the analytical and clinical validity of the digital measurements was investigated, without relying solely on correlation with clinical assessments, and it was demonstrated that the smartwatch algorithms developed using data from a Dutch population were transferable to a different study population and within a different healthcare setting. Overall, most V-scores were well generalized between the two datasets with the exception of V-tremor, which may be due to the differences in tremor severity between the two populations.

This study has shown that wearable devices can provide reliable and objective data from continuous monitoring of motor signs and symptoms in patients’ daily lives. Importantly, the findings demonstrated the effect of aggregating single-sensor–derived features into composite V-scores to build more reliable signals that correlated with clinical observations. Because this study was designed to establish proof of principle, the findings are preliminary in nature and will be used to inform future studies. In particular, this system allows collection of passive and active data, which can be integrated to develop reliable digital biomarkers. In addition, inclusion of a less intensive VME schedule is likely to be more suitable for long-term monitoring, and fine-tuning of medication tagging and patient engagement with the device are likely to contribute to more precise measurements in naturalistic settings.

## Methods

### Study design

This prospective, observational study was conducted at Juntendo University Hospital (Tokyo, Japan). Adult patients with PD currently being treated with levodopa, who were experiencing wearing off, and who required hospitalization to undergo monitoring of motor symptoms in accordance with routine clinical practice were recruited. Such patients included those with severe symptom fluctuations who required evaluation of device-aided therapy or treatment optimization.

The study protocol (SWJ-001, Version 2.0) was reviewed and approved by the Juntendo University Hospital Ethics Committee (#19-030, April 19, 2019), and the study was conducted in accordance with all applicable laws and regulations, including the ethical principles as outlined in the Declaration of Helsinki 1964 and its later amendments, Ethical Guidelines for Medical and Health Research Involving Human Subjects (the Ministry of Education, Culture, Sports, Science and Technology and the Ministry of Health, Labour and Welfare), and the International Council for Harmonisation E6 Good Clinical Practice: Consolidated Guideline. Written informed consent from patients or legal representatives was required before participation in any study procedures. The first patient was enrolled on July 30, 2019, and the study was completed on March 18, 2021.

The study comprised two outpatient visits at the beginning and end of the study, an optional 1-month home-based pre-hospitalization period, a 5-day inpatient assessment period, and a 1-month home-based post-hospitalization period. Patients were asked to wear the Verily Study Watch on their preferred arm regardless of the dominant or affected side for up to 23 h/day during the study and to charge and upload data from the device each day. All medical decisions during the study were conducted at the clinician’s discretion and in accordance with standard medical care. Only data for the inpatient and post-hospitalization home-based assessments are reported here.

### Study population

Patients aged 20 years or more with PD diagnosed in accordance with the 2018 Movement Disorder Society diagnostic criteria^28^ and of at least 5 years’ duration (to exclude other Parkinsonian syndromes) were enrolled in the study. At the time of informed consent, patients were being treated with oral levodopa with or without other medications and, in the opinion of the investigator, were capable of understanding and complying with protocol requirements, including adherence to the smartwatch procedures and performing simple, standardized motor tasks. Patients had to satisfy the following criteria: (1) have a clear-cut and robust response to levodopa, historically and presently, based on medical history and/or a formal clinical OFF- and ON-medication examination; (2) be experiencing motor fluctuations, with at least one troubling motor “OFF” period each day (with or without dyskinesia), as determined by the investigator using the MDS-UPDRS; and (3) be scheduled for at least 5 days of hospitalization as part of routine clinical care for an in-depth evaluation of motor function, with or without other therapeutic intervention.

The main exclusion criteria were allergy to nickel or metal jewelry; cognitive impairment or any medical condition that would interfere with interpretation of Parkinsonian motor symptoms; or in the opinion of the investigator, be at risk of harm when performing structured tasks at home or be ineligible for any other reason.

### Study protocol and virtual motor examination

#### Study watch

Wearers received minimal information from the smartwatch, which was intended for data collection only. The device displayed the date and time; wearers received on-device reminders to conduct the VME, used buttons to stop and start study tasks during the VME (Fig. 1), and could use the device to tag medication (Supplementary Fig. 3). Encrypted data were securely uploaded to a cloud platform using a USB charging cradle and wireless connectivity bridge (Study Hub). The device sensors captured movement (3-axis accelerometer and 3-axis gyroscope), pulse (photoplethysmography), and environmental conditions (ambient pressure, temperature, and light).

#### Study procedures

Assessments were conducted as outlined in Fig. 2. Motor function was assessed during the inpatient assessment by a neurologist using the Japanese version of the MDS-UPDRS Part III^6^ (motor examination) on Days 2, 3, and 5 according to a standard protocol. On Day 2, the MDS-UPDRS Part III was conducted four times, twice during an OFF state and twice during an ON state. On Day 3, it was conducted twice, once during the worst OFF state (12 h from the last levodopa dose) and once during the best ON state (1 h after an intravenous levodopa dose). On Day 3, the medical examination and clinical assessments were videotaped for review and scored by three independent neurologists. On Day 5, the MDS-UPDRS Part III was conducted twice, once during an OFF state and once during an ON state.

During each assessment period, the smartwatch VME application led patients through seven structured motor tasks twice daily at approximately the same times each day (Fig. 1). The VME assessment was programmed into the smartwatch for each patient at the first outpatient visit at times corresponding to when they were typically in the OFF (wearing off) or ON (i.e., when levodopa medication was working well) medication states. Core motor features were segregated temporally by digital tagging by the clinician during the inpatient examinations, and patients were asked to rate their performance on each VME task in five grades. During the inpatient assessment, the VME was conducted immediately after the MDS-UPDRS Part III when relevant. Patients were also asked to use the smartwatch to tag each time they took a dose of levodopa medication during the study; the timing of dosing was as prescribed and was not changed for this study.

Patient-reported health status and quality of life were assessed at enrollment and the exit visit using the Japanese paper-based version of the PDQ-39 (total scores from perfect to worst health [0–100])^29^, and ADL was assessed using the MDS-UPDRS Part II^6^ (at enrollment and the exit visit) and the Schwab and England ADL scale^30^ (weekly during each assessment period). Device use was evaluated via a questionnaire designed to assess satisfaction with the smartwatch and hub, including the use of its features, patient ease of use, and reasons for non-compliance, if present. The questions included: (1) study device: helpfulness of the instruction booklet, ease of setup, and ease of charging/syncing; (2) satisfaction: device comfort, device appearance, ease of cleaning, frequency of cleaning, desire to wear the device for longer; (3) frequency of use: main reason for not wearing the device every day when applicable; (4) ease of use of device buttons; (5) ease of use during the study procedure: ability to tag medication use on the device, ability to start and end tasks using the device.

### Development of digital measurements

The reference scores for patients’ motor function were assessed on Day 3 of the inpatient assessment using the MDS-UPDRS Part III and were calculated from the average of neurologist ratings (MDS-UPDRS consensus scores from three video raters).

**Figure 7:**
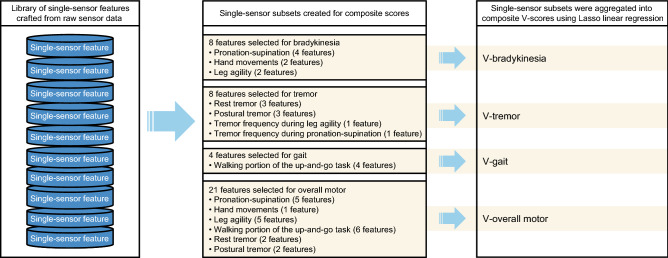
Development of composite V-scores from single-sensor–derived features. *V-score* machine-learned composite sensor scores for each motor feature.

Two types of digital measurements were developed: single-sensor–derived features and composite scores (V-scores) (Fig. 7). First, a library of single-sensor features was developed from the literature^16,31–36^ and interviews with expert neurologists to capture meaningful signals from tri-axial accelerometer and gyroscope data collected during the in-clinic MDS-UPDRS examinations and the home-based VMEs. After extraction of features with high signal-to-noise ratios and those that were highly correlated, a subset was then included in the final model (with correlation to MDS-UPDRS consensus scores as the outcome variable) using a recursive feature elimination procedure. Second, Lasso linear regression models adjusted for MDS-UPDRS Part III consensus scores were used to aggregate the subset of sensor features into V-scores; Lasso penalty ensured model sparsity and limited the number of features for inclusion. Twenty-seven single-sensor features were selected for inclusion in the final machine-learning models to create V-scores for bradykinesia, tremor, gait, and overall motor features. Of the 27 single-sensor features (Fig. 7), eight were clinically intuitive and are reported here. Machine-learning models were trained to estimate the clinician-rated MDS-UPDRS Part III consensus scores using data collected from the PPP cohort in the Netherlands^19^; the models were fine-tuned with data from 10 patients in the current study, and correlations between MDS-UPDRS Part III V-scores and neurologist-rated consensus scores were consistent between the two populations for all V-scores, except V-tremor (Supplementary Table 7).

### Evaluation of digital measurements

Analytical validity of smartwatch digital measurements was explored by assessing correlation between MDS-UPDRS Part III sensor scores and the video-based neurologist-rated MDS-UPDRS Part III consensus scores on Day 3 of the inpatient assessment and the test–retest reliability of MDS-UPDRS Part III sensor scores on Day 2 of the inpatient assessment. Clinical validity of smartwatch digital measurements was assessed by evaluating whether the digital measurements were able to detect differences that were caused by levodopa. The degree of difference between ON and OFF states that a digital measurement was able to detect is reported as the “levodopa effect size”. Levodopa effect sizes from the worst OFF state and the best ON state were calculated during the investigator-supervised MDS-UPDRS Part III examination and the unsupervised VME on Day 3 of the inpatient assessment, and test–retest reliability of VME sensor scores was assessed during the post-hospitalization home-based period. Other assessments included correlation between home-based VME sensor scores and patient self-reported functioning (Schwab and England ADL scale, MDS-UPDRS Part II), health status, and quality of life (PDQ-39 summary index) assessed during the home-based period, and correlation between neurologist-rated MDS-UPDRS Part III consensus scores and the unsupervised VME assessed on Day 3 of the inpatient assessment.

Correlation analyses were conducted using Spearman rank correlation, and test–retest reliability was conducted using the ICC. Effect sizes were calculated as standardized mean differences (Cohen’s d). Confidence intervals were generated by bootstrapping with 1000 resampling iterations. Unless otherwise specified, the average of all measurements for a given patient was used for sensor measures collected at multiple time points. Missing data were not imputed. Figures and statistical analyses were generated using Python programming language, using the SciPy, Matplotlib, and Seaborn libraries.

## Supplementary Information

Supplementary Information.

## Data Availability

The datasets generated during and/or analysed during the current study are available from the corresponding author on reasonable request.
