# Supplementary material for: Analytical and clinical validity of wearable, multi-sensor technology for assessment of motor function in patients with Parkinson’s disease in Japan
Source: Sci Rep. 2023 Mar 14;13:3600. doi: 10.1038/s41598-023-29382-6 (PMC10015076; doi:10.1038/s41598-023-29382-6)
Supplement: Supplementary file 1 — Supplementary Information. [file 41598_2023_29382_MOESM1_ESM.pdf]

SUPPLEMENTARY MATERIAL

Analytical and clinical validity of wearable, multi-sensor technology for assessment of motor function in patients with Parkinson’s disease in Japan

AUTHORS

Genko Oyama[1,*], Maximilien Burq[2,*], Taku Hatano[1], William J. Marks, Jr[2], Ritu Kapur[2], Jovelle Fernandez[3], Keita Fujikawa[3], Yoshihiko Furusawa[3], Keisuke Nakatome[3], Erin Rainaldi[2], Chen Chen[2], King Chung Ho[2], Takashi Ogawa[1], Hikaru Kamo[1], Yutaka Oji[1], Haruka Takeshige-Amano[1], Daisuke Taniguchi[1], Ryota Nakamura[1], Fuyuko Sasako[1], Shinichi Ueno[1], Kenta Shiina[1], Anri HAttori[1], Noriko Nishikawa[1], Mayu Ishiguro[1], Shinji Saiki[1], Ayako Hayashi[3], Masatoshi Motohashi[3], Nobutaka Hattori

* These authors contributed equally to the work

AUTHORS’ AFFILIATIONS

[1] Department of Neurology, Juntendo University Faculty of Medicine, 2-1-1, Hongo, Bunkyo-ku, Tokyo 113-8421, Japan

[2] Verily Life Sciences, 269 East Grand Avenue, South San Francisco, CA, USA

[3] Takeda Pharmaceutical Company Limited, 2 Chome-1-1 Nihonbashihoncho, Chuo City, Tokyo 103-0023, Japan

CORRESPONDING AUTHOR

Genko Oyama

Department of Neurology, Juntendo University Faculty of Medicine, 2-1-1, Hongo, Bunkyo-ku, Tokyo 113-8421, Japan

Phone: +81-3-3813-3111; Fax: +81-3-5800-0547

Email: g_oyama@juntendo.ac.jp

LIST OF TABLES AND FIGURES

Supplementary Fig. 1 Patient adherence with the virtual motor examination (VME).

Supplementary Fig. 2 Patient ratings for ease of use and comfort.

Supplementary Fig. 3 Study Watch face showing medication tagging

Supplementary Table 1. Patients included in the analyses

Supplementary Table 2. Analytical validity of digital measurements from single-sensor–derived features and composite V-scores: correlation between MDS-UPDRS Part III sensor scores and neurologist-rated consensus scores and test-retest reliability during the inpatient assessment.

Supplementary Table 3. Clinical validity of digital measurements from single-sensor–derived features and composite V-scores as pharmacodynamic biomarkers: levodopa effect sizes calculated using sensor data collected during the supervised MDS-UPDRS Part III and unsupervised VME on Day 3 of the inpatient assessment.

Supplementary Table 4. Test-retest reliability of the VME from single-sensor–derived features and composite V-scores during the post-hospitalization home-based assessment.

Supplementary Table 5. Correlation between VME sensor scores during the post-hospitalization home-based assessment and patient self-reported functioning for single-sensor–derived features and composite V-scores

Supplementary Table 6. Correlation between neurologist-rated MDS-UPDRS Part III consensus scores collected on Day 3 of the inpatient assessment and VME sensor scores collected on Day 3 of the inpatient assessment and during the home-based assessment for single-sensor–derived features and composite V-scores

Supplementary Table 7. Analytical validity of digital measurements from composite V-scores: Spearman rank correlation between MDS-UPDRS Part III sensor scores and neurologist-rated consensus scores for patients from the Study Watch study in the Netherlands[1] and in Japan

Supplementary Fig. 1 Patient adherence with the virtual motor examination (VME). a Time taken to complete the VME by medication state; **b** for incomplete VMEs, the percentage of tasks that were skipped, by medication state.

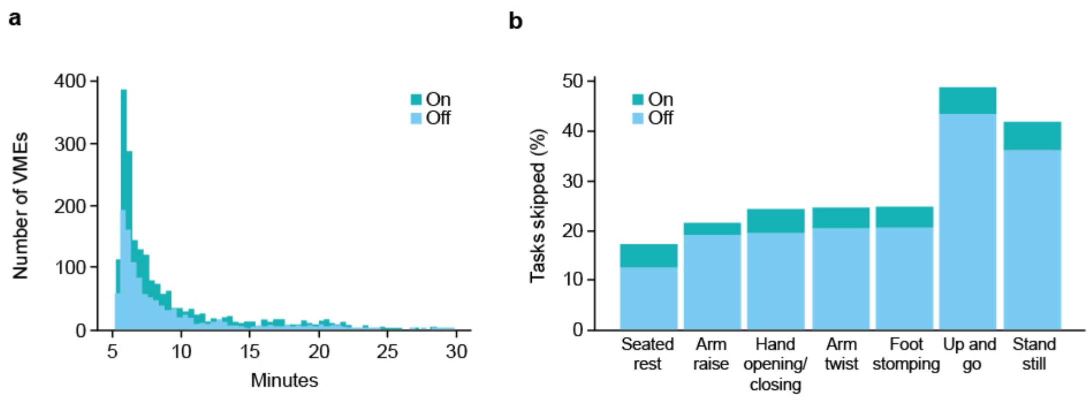

Supplementary Fig. 2 Patient ratings for ease of use and comfort. A total of 92 patients responded to the survey.

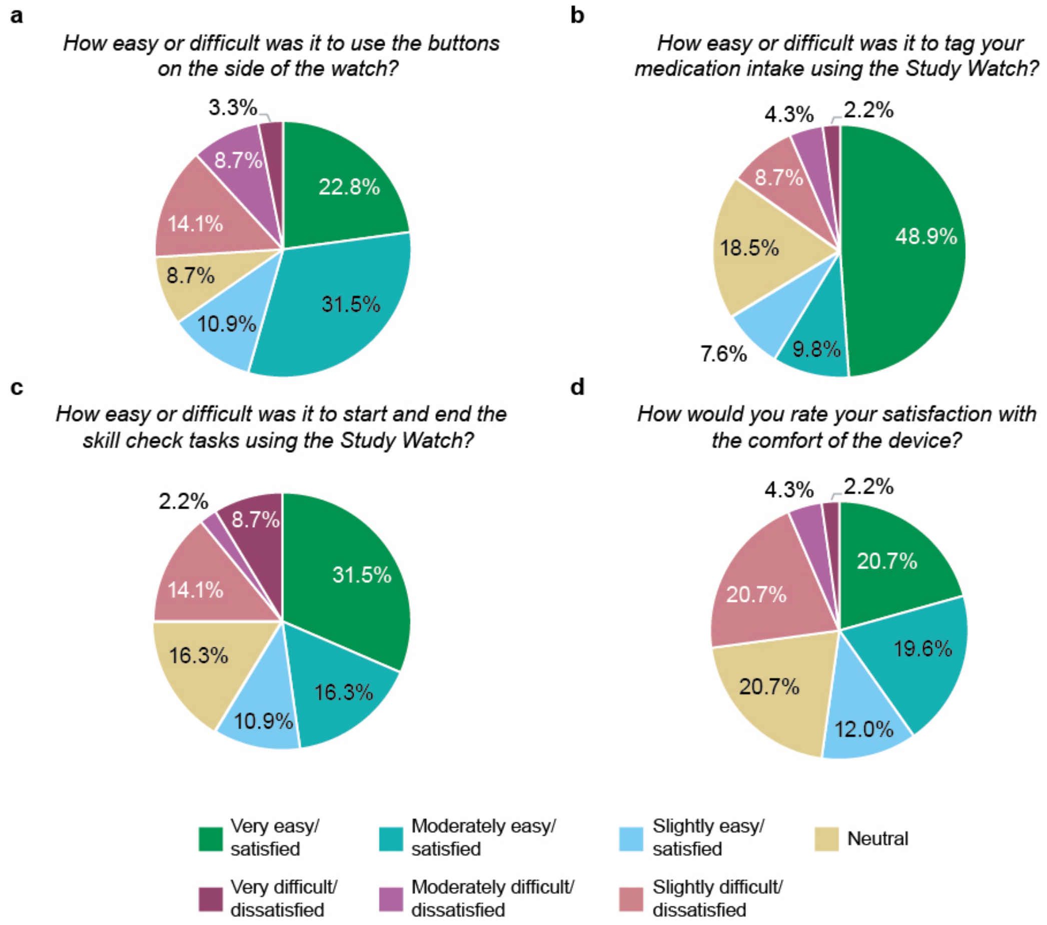

Supplementary Fig. 3 Study Watch face showing medication tagging.

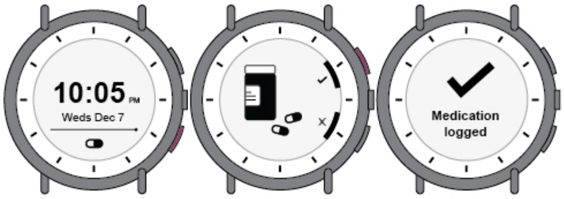

Supplementary Table 1. Patients included in the analyses.

Study period[a]	Any clinical or VME data	Valid[b] VMEs		Two valid VMEs and efficient[c] medication tagging	
	Number of patients	Number of patients	Number of VMEs	Number of patients	Mean (SD), days
Period 2 – inpatient assessment	96	96	1,695	79	4.1 (2.7)
Period 3 – home-based post-hospitalization assessment	89	84	2,396	66	7.9 (6.9)

[a]Period 2 VMEs were conducted twice daily for 5 days; Period 3 VMEs were conducted twice daily for 1 month.
[b]A VME was considered valid if sufficient data could be extracted to derive smartwatch sensor measurements.
[c]VMEs with efficient medication tagging were those cases where medication was tagged and where a patient's OFF/ON medication state could be inferred using predefined thresholds (ON was defined as a VME conducted at least 30 minutes after and within 4 hours of the last medication tag; OFF was a VME conducted 6 hours after the last medication tag and within 24 hours of the next medication tag; Unknown was when the VME took more than 30 minutes to execute).
SD standard deviation, *VME* virtual motor examination.

Supplementary Table 2. Analytical validity of digital measurements from single-sensor–derived features and composite V-scores: correlation between MDS-UPDRS Part III sensor scores and neurologist-rated consensus scores and test-retest reliability during the inpatient assessment.[a,b]

Measure	n	Spearman correlation, r (95% CI)[c]	Test-retest reliability, ICC (95% CI)[d]
**Single-sensor features**			
Bradykinesia			
Upper bradykinesia amplitude, degrees	89	-0.44 (-0.54, -0.35)	0.60 (0.43, 0.76)
Upper bradykinesia rate, units/s	89	-0.57 (-0.66, -0.48)	0.54 (0.29, 0.78)
Lower bradykinesia amplitude, m/s	91	-0.48 (-0.58, -0.38)	0.57 (0.44, 0.68)
Lower bradykinesia rate, units/s	91	-0.37 (-0.48, -0.25)	0.29 (0.10, 0.48)
Gait			
Gait cadence, steps/s	90	-0.34 (-0.46, -0.23)	0.64 (0.48, 0.77)
Arm swing acceleration, m/s^2	90	-0.61 (-0.68, -0.53)	0.63 (0.49, 0.75)
Tremor			
Postural tremor amplitude, m	91	0.13 (-0.01, 0.25)	0.18 (-0.01, 0.43)
Postural tremor acceleration, m/s^2	91	0.28 (0.16, 0.40)	0.53 (0.38, 0.67)
Rest tremor amplitude, m	91	-0.07 (-0.19, 0.03)	0.22 (0.07, 0.48)
Rest tremor acceleration, m/s^2	91	0.07 (-0.05, 0.20)	0.58 (0.45, 0.71)
**Composite V-scores**			
V-bradykinesia	89	0.63 (0.55, 0.70)	0.72 (0.61, 0.82)
V-gait	89	0.55 (0.47, 0.63)	0.57 (0.43, 0.70)
V-tremor	89	0.41 (0.28, 0.52)	0.65 (0.51, 0.74)
V-overall motor	86	0.70 (0.62, 0.76)	0.67 (0.52, 0.79)

[a]Consensus scores for the MDS-UPDRS Part III examination on Day 3 of the inpatient assessment were calculated using videotaped ratings from three neurologists.
[b]The averages of all scores for the OFF and ON states on Day 3 were combined for each measure.
[c]Spearman rank correlation was considered weak for coefficients <0.3, moderate for coefficients 0.3-0.6, and strong for coefficients >0.6.
[d]Test-retest reliability was computed from MDS-UPDRS Part III sensor scores on Day 2, on which the MDS-UPDRS examination was administered twice within a short period of time; test-retest reliability was considered poor for ICCs <0.5, average for ICCs 0.5-0.75, good for ICCs >0.75-0.9, and excellent for ICCs >0.9.
CI confidence interval, *ICC* intraclass correlation coefficient, *MDS-UPDRS* Movement Disorder Society-sponsored revision of the Unified Parkinson's Disease Rating Scale, *PD* Parkinson's disease, *V-score* machine-learned composite sensor scores for each motor feature.

Supplementary Table 3. Clinical validity of digital measurements from single-sensor–derived features and composite V-scores as pharmacodynamic biomarkers: levodopa effect sizes calculated using sensor data collected during the supervised MDS-UPDRS Part III and unsupervised VME on Day 3 of the inpatient assessment.

Measure	Levodopa effect sizes[a] (OFF – ON states)			
	n	Supervised MDS-UPDRS Part III[b]	n	Unsupervised VME[b]
**Single-sensor features**				
Bradykinesia				
Upper bradykinesia amplitude, degrees	76	-0.33 (-0.50, -0.17)	87	-0.78 (-0.95, -0.65)
Upper bradykinesia rate, units/s	76	-0.50 (-0.72, -0.34)	87	-0.36 (-0.53, -0.21)
Lower bradykinesia amplitude, m/s	88	-0.61 (-0.78, -0.48)	89	-0.67 (-0.81, -0.55)
Lower bradykinesia rate, units/s	88	-0.18 (-0.34, -0.02)	89	0.00 (-0.18, 0.16)
Gait				
Gait cadence, steps/s	78	-0.45 (-0.67, -0.28)	78	-0.38 (-0.54, -0.23)
Arm swing acceleration, m/s^2	78	-1.14 (-1.35, -0.97)	78	-1.39 (-1.58, -1.24)
Tremor				
Postural tremor amplitude, m	88	-0.06 (-0.24, 0.11)	89	-0.76 (-0.94, -0.59)
Postural tremor acceleration, m/s^2	88	0.25 (0.10, 0.46)	89	0.08 (-0.07, 0.22)
Rest tremor amplitude, m	88	-0.58 (-0.83, -0.42)	89	-0.62 (-0.84, -0.45)
Rest tremor acceleration, m/s^2	88	-0.21 (-0.41, -0.06)	89	-0.17 (-0.36, -0.01)
**Composite V-scores**				
V-bradykinesia	73	0.83 (0.66, 1.06)	83	0.79 (0.65, 0.97)
V-gait	61	1.20 (1.02, 1.44)	70	1.37 (1.19, 1.56)
V-tremor	76	0.51 (0.36, 0.71)	87	0.56 (0.45, 0.67)
V-overall motor	53	1.44 (1.21, 1.80)	66	1.36 (1.19, 1.62)

[a]Effect size was calculated using Cohen's d. Effect sizes of 0.2 were considered small, 0.5 were considered medium, 0.8 large, and >1.2 very large.
[b]Smartwatch sensor data were collected during the investigator-supervised MDS-UPDRS Part III examination and during the unsupervised VME on Day 3 of the inpatient assessment; the averages of all scores on Day 3 were combined for each measure.
MDS-UPDRS Movement Disorder Society-sponsored revision of the Unified Parkinson's Disease Rating Scale, *V-score* machine-learned composite sensor scores for each motor feature, *VME* virtual motor examination.

Supplementary Table 4. Test-retest reliability of the VME from single-sensor–derived features and composite V-scores during the post-hospitalization home-based assessment.

Measure	n	Day-over-day, ICC (95% CI)[a]	Week-over-week, ICC (95% CI)[a]
**Single-sensor features**			
Bradykinesia			
Upper bradykinesia amplitude, degrees	57	0.77 (0.74, 0.81)	0.83 (0.74, 0.89)
Upper bradykinesia rate, units/s	57	0.74 (0.66, 0.81)	0.86 (0.76, 0.93)
Lower bradykinesia amplitude, m/s	57	0.49 (0.42, 0.55)	0.62 (0.44, 0.77)
Lower bradykinesia rate, units/s	57	0.35 (0.27, 0.43)	0.69 (0.58, 0.78)
Gait			
Gait cadence, steps/s	56	0.52 (0.46, 0.58)	0.70 (0.59, 0.8)
Arm swing acceleration, m/s^2	56	0.55 (0.49, 0.6)	0.73 (0.63, 0.82)
Tremor			
Postural tremor amplitude, m	58	0.04 (0.01, 0.17)	0.09 (-0.01, 0.52)
Postural tremor acceleration, m/s^2	58	0.46 (0.39, 0.53)	0.55 (0.41, 0.7)
Rest tremor amplitude, m	62	0.24 (0.15, 0.34)	0.62 (0.35, 0.82)
Rest tremor acceleration, m/s^2	62	0.39 (0.31, 0.46)	0.63 (0.48, 0.75)
**Composite V-scores**			
V-bradykinesia	54	0.66 (0.61, 0.71)	0.77 (0.68, 0.84)
V-gait	54	0.57 (0.51, 0.62)	0.75 (0.66, 0.82)
V-tremor	57	0.55 (0.47, 0.63)	0.59 (0.41, 0.74)
V-overall motor	52	0.66 (0.61, 0.71)	0.79 (0.7, 0.86)

[a]Day-over-day and week-over-week test-retest reliability for daily and weekly aggregated VME sensor scores, respectively, was assessed by taking the daily measurements and weekly averages of all scores for the OFF and ON states during the post-hospitalization period. Test-retest reliability was considered poor for ICCs <0.5, average for ICCs 0.5-0.75, good for ICCs >0.75-0.9, and excellent for ICCs >0.9.

CI confidence interval, *ICC* intraclass correlation coefficient, *V-score* machine-learned composite sensor scores for each motor feature, *VME* virtual motor examination.

Supplementary Table 5. Correlation between VME sensor scores during the post-hospitalization home-based assessment and patient self-reported functioning for single-sensor–derived features and composite V-scores.[a]

Clinical measurement	Digital measurement	OFF state		ON state	
		n	Spearman correlation	n	Spearman correlation
**MDS-UPDRS Part II**	Upper bradykinesia amplitude, degrees	60	-0.20	68	-0.32
	Upper bradykinesia rate, units/s	60	0.09	68	0.11
	Lower bradykinesia amplitude, m/s	62	0.00	69	-0.02
	Lower bradykinesia rate, units/s	61	0.27	69	0.16
	Gait cadence, steps/s	63	-0.29	69	-0.26
	Arm swing acceleration, m/s^2	63	-0.31	69	-0.16
	Postural tremor amplitude, m	63	0.11	70	0.05
	Postural tremor acceleration, m/s^2	63	0.17	70	-0.01
	Rest tremor amplitude, m	63	0.24	72	0.04
	Rest tremor acceleration, m/s^2	63	0.23	72	-0.01
	**V-bradykinesia**	**61**	**0.23**	**68**	**0.29**
	**V-gait**	**61**	**0.33**	**68**	**0.23**
	**V-tremor**	**61**	**0.05**	**68**	**0.03**
	**V-total motor score**	**59**	**0.35**	**67**	**0.38**
**PDQ-39 summary index**	Upper bradykinesia amplitude, degrees	60	-0.16	68	-0.16
	Upper bradykinesia rate, units/s	60	0.11	68	0.17
	Lower bradykinesia amplitude, m/s	62	0.02	69	0.07
	Lower bradykinesia rate, units/s	61	0.22	69	0.08
	Gait cadence, steps/s	63	-0.19	69	-0.11
	Arm swing acceleration, m/s^2	63	-0.33	69	-0.05
	Postural tremor amplitude, m	63	-0.05	70	-0.13
	Postural tremor acceleration, m/s^2	63	0.12	70	-0.11
	Rest tremor amplitude, m	63	0.15	72	-0.08
	Rest tremor acceleration, m/s^2	63	0.18	72	-0.08
	**V-bradykinesia**	**61**	**0.17**	**68**	**0.21**
	**V-gait**	**61**	**0.32**	**68**	**0.13**
	**V-tremor**	**61**	**0.19**	**68**	**0.08**
	**V-total motor score**	**59**	**0.25**	**67**	**0.21**
**Schwab and England ADL**	Upper bradykinesia amplitude, degrees	60	0.10	68	0.25
	Upper bradykinesia rate, units/s	60	-0.07	68	-0.12
	Lower bradykinesia amplitude, m/s	62	-0.03	69	0.08
	Lower bradykinesia rate, units/s	61	-0.16	69	-0.12
	Gait cadence, steps/s	63	0.16	69	0.09
	Arm swing acceleration, m/s^2	63	0.16	69	0.19
	Postural tremor amplitude, m	63	-0.08	70	0.19
	Postural tremor acceleration, m/s^2	63	-0.11	70	0.17
	Rest tremor amplitude, m	63	-0.07	72	-0.07
	Rest tremor acceleration, m/s^2	63	-0.06	72	-0.07
	**V-bradykinesia**	**61**	**-0.03**	**68**	**-0.23**
	**V-gait**	**61**	**-0.13**	**68**	**-0.23**
	**V-tremor**	**61**	**0.02**	**68**	**0.03**
	**V-total motor score**	**59**	**-0.14**	**67**	**-0.34**

[a]Spearman rank correlation was assessed for VME data collected during the post-hospitalization home-based assessment and clinical data collected at enrollment; the averages of all scores available for each measure were combined for these analyses (PDQ-39 and MDS-UPDRS Part II: at enrollment and at the exit visit; Schwab and England ADL: weekly during the post-hospitalization home-based assessment; symptom scores: aggregated weekly scores from the post-hospitalization home-based assessment. Pearson correlation was considered weak for coefficients <0.3, moderate for coefficients 0.3-0.6, and strong for coefficients >0.6.

ADL Activities of Daily Living, *MDS-UPDRS* Movement Disorder Society-sponsored revision of the Unified Parkinson's Disease Rating Scale, *PDQ-39* 39-item Parkinson's Disease Questionnaire, *V-score* machine-learned composite sensor scores for each motor feature, *VME* virtual motor examination.

Supplementary Table 6. Correlation between neurologist-rated MDS-UPDRS Part III consensus scores collected on Day 3 of the inpatient assessment and VME sensor scores collected on Day 3 of the inpatient assessment and during the home-based assessment for single-sensor–derived features and composite V-scores.[a]

Measure	Unsupervised VME Inpatient assessment		Unsupervised VME Home-based assessment	
	n	Spearman r (95% CI)[b]	n	Spearman r (95% CI)[b]
**Single-sensor features**				
Bradykinesia				
Upper bradykinesia amplitude, degrees	57	-0.24 (-0.39, -0.1)	77	-0.22 (-0.35, -0.08)
Upper bradykinesia rate, units/s	57	-0.36 (-0.5, -0.21)	77	-0.26 (-0.38, -0.11)
Lower bradykinesia amplitude, m/s	57	-0.33 (-0.47, -0.17)	78	-0.24 (-0.37, -0.11)
Lower bradykinesia rate, units/s	57	-0.36 (-0.51, -0.18)	77	-0.24 (-0.36, -0.1)
Gait				
Gait cadence, steps/s	56	-0.18 (-0.32, -0.04)	78	-0.29 (-0.42, -0.16)
Arm swing acceleration, m/s^2	56	-0.24 (-0.39, -0.1)	78	-0.24 (-0.36, -0.12)
Tremor				
Postural tremor amplitude, m	57	0.11 (-0.04, 0.26)	79	0.03 (-0.1, 0.16)
Postural tremor acceleration, m/s^2	57	0.26 (0.1, 0.41)	79	0.1 (-0.03, 0.22)
Rest tremor amplitude, m	58	0.13 (-0.04, 0.3)	78	0.0 (-0.12, 0.12)
Rest tremor acceleration, m/s^2	58	0.18 (0.0, 0.34)	78	0.04 (-0.1, 0.16)
**Composite V-scores**				
V-bradykinesia	57	0.37 (0.23, 0.48)	75	0.23 (0.08, 0.36)
V-gait	56	0.27 (0.12, 0.42)	76	0.22 (0.1, 0.35)
V-tremor	57	0.34 (0.18, 0.5)	77	0.10 (-0.04, 0.22)
V-overall motor	56	0.39 (0.28, 0.5)	73	0.30 (0.17, 0.42)

[a]Scores for the OFF and ON states were combined for these analyses; average values for each patient were analyzed.
[b]Spearman rank correlation was considered weak for coefficients <0.3, moderate for coefficients 0.3-0.6, and strong for coefficients >0.6.
CI confidence interval, *MDS-UPDRS* Movement Disorder Society-sponsored revision of the Unified Parkinson's Disease Rating Scale, *V-score* machine-learned composite sensor scores for each motor feature, *VME* virtual motor examination.

Supplementary Table 7. Analytical validity of digital measurements from composite V-scores: Spearman rank correlation between MDS-UPDRS Part III sensor scores and neurologist-rated consensus scores for patients from the Study Watch study in the Netherlands[1] and in Japan.

	Dutch population		Japanese population[a,b]	
Composite V-scores	n	r (95% CI)	n	r (95% CI)
V-bradykinesia	97	0.65 (0.51, 0.75)	89	0.63 (0.55, 0.70)
V-gait	80	0.52 (0.33, 0.68)	89	0.55 (0.47, 0.63)
V-tremor	79	0.77 (0.66, 0.85)	89	0.41 (0.28, 0.52)
V-posture	75	0.39 (0.17, 0.58)	90	0.35 (0.24, 0.45)
V-overall motor	75	0.63 (0.48, 0.76)	86	0.70 (0.62, 0.76)

[a]Consensus scores for the MDS-UPDRS Part III examination on Day 3 of the inpatient assessment were calculated using videotaped ratings from three neurologists.
[b]The averages of all scores for the OFF and ON states on Day 3 were combined for each measure.
1. Burq, M. et al. Virtual exam for Parkinson's disease enables frequent and reliable remote measurements of motor function. *NPJ Digit Med* **5**, 65 (2022).
CI confidence interval, *MDS-UPDRS* Movement Disorder Society-sponsored revision of the Unified Parkinson's Disease Rating Scale, *V-score* machine-learned composite sensor scores for each motor feature.
